# A role for neuropeptides in innate immune inflammation of the nose

**DOI:** 10.1186/2045-7022-5-S4-O2

**Published:** 2015-06-26

**Authors:** Olivia Larsson, Lotta Tengroth, Yuan Xu, Susanna Kumlien Georen, Lars-Olaf Cardell

**Affiliations:** 1 Karolinska Institutet, Division of ENT Diseases, CLINTEC, Stockholm, Sweden; 2 Karolinska Institutet and Karolinska University Hospital, Division of ENT Diseases, CLINTEC, Stockholm, Sweden

## Background

The airway epithelium constitutes the first line of defense in the protection against invading pathogens. It acts as a barrier, but it is also is a major source of early released inflammatory mediators, which help shape the inflammatory response. Neuropeptides, such as substance P (SP), have long been considered to be early contributors to the inflammatory response, causing pain hypersensitivity and vasodilation, as well as activation and infiltration of various immune cells. Toll-like receptor 7 (TLR7) is found on the epithelial cells and is known to be activated by viruses. The present study has investigated the relationship between TLR7 activation/expression and SP release/stimulation.

## Method

Human nasal epithelial cells (HNEC) were obtained through nasal brushing of 6 healthy donors. The cells were cultured until passage 4 and thereafter stimulated with the TLR7 agonists R-837 or R-848 (1, 5 or 10μg/ml) for 15min, 30 min or 4h. The subsequent release of SP was analyzed with EIA. In addition, HNECs were stimulated with SP (10, 50 or 100nM) for 30 minutes in the presence or absence of NK-1 antagonist Aprepitant. Expression of Toll-like receptors was then determined using flow cytometry.

## Results

HNECs produced substance P in a concentration-dependent manner in response to both R-837 and R-848. Increased levels of SP were detected already after 15 minutes, and increased successively over time. SP stimulation increased not only the TLR7 expression in HNECs, but also expression of TLR1, 4 and 9 on these cells. Aprepitant effectively blocked this response.

## Conclusion

The presented results suggest a role for SP in modulating the local innate immune response in the nose.

